# Early prevention of radial artery occlusion *via* distal transradial access for primary percutaneous coronary intervention

**DOI:** 10.3389/fcvm.2022.1071575

**Published:** 2022-11-30

**Authors:** Yujie Wang, Zijing Liu, Yongxia Wu, Zixuan Li, Yuntao Wang, Senhu Wang, Rong Xu, Libin Zhang, Yuping Wang, Jincheng Guo

**Affiliations:** Division of Cardiology, Beijing Luhe Hospital, Capital Medical University, Beijing, China

**Keywords:** radial artery occlusion, ST-segment elevation myocardial infarction, primary percutaneous coronary intervention, distal radial access, conventional transradial access

## Abstract

**Background:**

Conventional transradial access (TRA) has been the preferred access for coronary intervention. Recently, distal radial access (DRA) is introduced as an alternative choice to reduce radial artery occlusion (RAO) risk. The study sought to assess the impact of DRA on early RAO using Doppler ultrasound in patients with ST-segment elevation myocardial infarction (STEMI) who underwent primary percutaneous coronary intervention (PCI).

**Methods:**

This is a prospective, single-center, open-label randomized clinical trial in which patients with indications for primary PCI from January 2022 to September 2022 were assigned to DRA or TRA group with 100 cases in each group. The primary endpoint was the incidence of forearm RAO, evaluated by Doppler ultrasound before discharge.

**Results:**

The rate of access success was comparable between the DRA and TRA groups (98.0 vs. 94.0%, *P* = 0.279). Compared with the TRA group, longer puncture time was observed in the DRA group [2.4 (1.7–4.2) min vs. 1.7 (1.4–2.3) min; *P* < 0.001] whereas the door-to-wire time was not delayed in primary PCI [71 (54–88) min vs. 64 (56–82) min, *P* = 0.103]. Shorter hemostasis time was required in the DRA group [3.1 (2.7–3.3) h vs. 6.2 (5.9–6.4) h; *P* < 0.001]. Significant reduction of the incidence of forearm RAO was observed in the DRA group (2.0 vs. 9.0%, *P* = 0.030). Local hematomas ≤ 5 cm was similar in both groups (4.0 vs. 6.0%, *P* = 0.516), while those > 5 cm were significantly more frequent in the TRA group (0 vs. 6.0%, *P* = 0.029).

**Conclusion:**

Distal radial access is associated with a comparable lower incidence of forearm RAO, shorter hemostasis time, and lower rate of vascular complications compared to TRA in primary PCI.

**Systematic review registration:**

[https://www.chictr.org.cn], identifier [ChiCTR2200061841].

## Introduction

Conventional transradial access (TRA) is strongly recommended as the standard approach for coronary angiography (CAG) and intervention, mainly in patients with acute ST-segment elevation myocardial infarction (STEMI) undergoing primary percutaneous coronary intervention (PCI) (1). In contemporary practice, approximately 50% of patients with STEMI have multivessel disease at the time of primary PCI, of which nearly 50% needed staged treatment of non-culprit lesions (2, 3). Therefore, maintenance of radial artery patency is of utmost importance for repeated coronary procedures. However, radial artery occlusion (RAO) remains the most frequent postprocedural complication of TRA, with incidence of 7.7% for early RAO within 24 h post-procedure and of 5.5% at 1 month (4). RAO also limits the usage of the same radial artery as a graft for coronary artery bypass grafting, or as the site for arteriovenous fistula in patients requiring hemodialysis.

Several strategies have been proposed and proven effective in maintaining radial artery patency including adequate procedural anticoagulation, low sheath to artery size ratio, patent hemostasis, the use of prophylactic ipsilateral ulnar artery compression and shorter post-procedure compression duration (5–8). Despite these recommendations, the contemporary “real-world” incidence of acute RAO continues to reach 5% (9).

Recently, distal radial access (DRA) through the anatomical snuffbox has received much attention due to its anatomic and physiological advantages over TRA, which yields potential for lower rates of forearm RAO and a shorter time required for hemostasis (10, 11). A unique pathophysiological study shows that simulated occlusion of the radial artery in the anatomical snuffbox did not affect forearm blood flow in contrast to simulated occlusion at the wrist level (12). Several randomized-control trials and meta-analyses have showed that DRA was associated with a substantial reduction of RAO (13–16). As shown in the DISCO RADIAL trial (17), DRA certainly may prolong the time to secure access but may increase the simplicity and the safety of the hemostasis phase without no specific impact on the procedural phase itself. However, most studies excluded STEMI patients. It is unclear whether the benefits of DRA can be extrapolated to STEMI patients undergoing primary PCI, which should be performed in a timely fashion.

In the present study, we performed a randomized trial to assess the efficacy and safety of DRA compared with TRA in patients undergoing primary PCI.

## Methods

### Study design and population

This prospective, single-center, open-label randomized clinical trial (ChiCTR2200061841) with a 1:1 allocation ratio enrolled 200 patients presenting with STEMI who underwent primary PCI at the department of cardiology, Beijing Luhe Hospital from January 2022 to September 2022. Patients aged > 18 years with an onset time < 12 h for primary PCI and the presence of palpable pulse in both the wrist and anatomical snuffbox were recruited in the study. The exclusion criteria were hemodynamic instability, previous coronary artery bypass graft (CABG), non-compliance with the study protocol, or refusal to give written informed consent. The enrolled participants were randomly separated into the DRA group (*n* = 100) and TRA group (*n* = 100) using closed envelopes. All procedures were performed by three interventional operators with extensive experience in TRA (more than 5 years) and a run-in phase of DRA (at least 200 procedures). This study was approved by the ethics committee of our hospital, and all patients provided written informed consent before randomization.

### Procedure

All patients were pretreated with a loading dose of 300 mg aspirin and a P2Y12 inhibitor (clopidogrel 600 mg or ticagrelor 180 mg) before primary PCI. Right access site was the first choice for both DRA and TRA group in our study.

**FIGURE 1:**
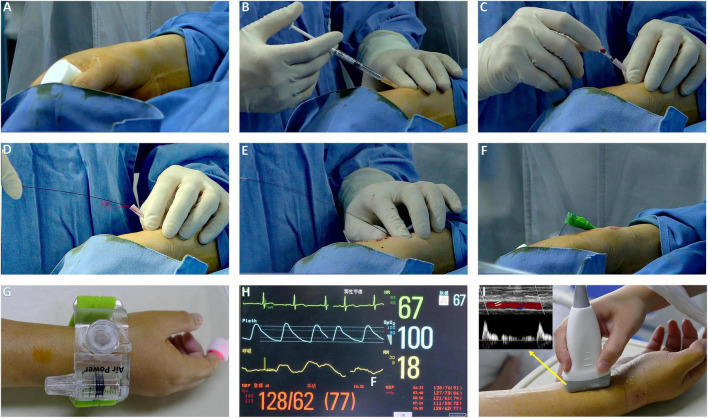
The procedure setup of DRA. **(A)** The patient arm is placed with its lateral side facing upward, hold a roll of gauze. **(B)** Local anesthesia. **(C)** Puncture DRA with 20 G plastic cannula. **(D)** Pulsatile blood flow. **(E)** Introduction of miniguide wire. **(F)** The sheath in place. **(G,H)** Hemostasis with air power device and index finger for pulse oximeter. **(I)** Evaluation of radial patency by Doppler ultrasound.

In the DRA group, the upper limb was placed along the patient’s body in a neutral position with a roll of gauze held in the hand to bring the distal radial artery to the surface (Figure 1A). The puncture site was located at anatomical snuffbox. After sterile preparation and subcutaneous injection of local anesthesia with 2% lidocaine (Figure 1B), the arterial puncture was performed successfully with a 20-gauge needle with plastic cannula (Figure 1C) and then a 0.025″ mini guidewire was carefully advanced (Figures 1D,E). If the mini guidewire failed to pass through, a soft-tipped 0.014″ percutaneous transluminal coronary angioplasty (PTCA) guide wire was advanced first, followed by advancing the venipuncture catheter, and then exchanged for a 0.025″ guidewire (18). Finally, a standard 6Fr sheath of 16-cm sheath was introduced (Terumo, Tokyo, Japan; Figure 1F). In cases of failure of radial artery cannulation, the alternative access site was left to the operator’s discretion. After completion of the procedure, patent haemostasias was achieved by a radial compression device for 2–3 h (Air Power, Shenzhen, China; Figures 1G,H). In case of bleeding or swelling with minor hematoma at puncture site, an additional 30 min to 1 h of compression was applied. Before discharge, radial artery patency was evaluated with Doppler ultrasound (Figure 1I).

**FIGURE 2:**
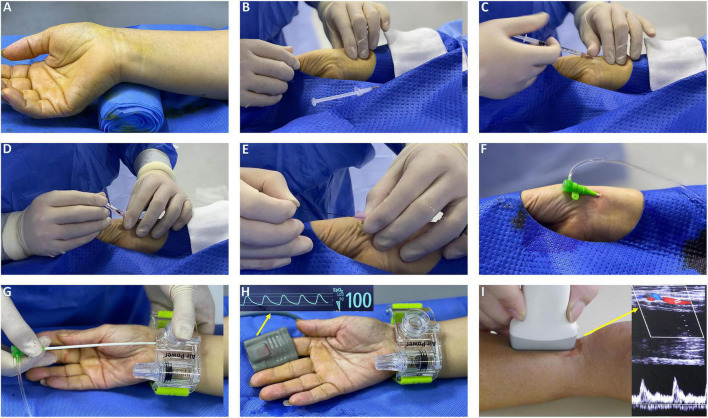
The procedure setup of TRA. **(A)** Hand position for radial artery puncture and rolled up towel under the dorsal wrist. **(B)** Palpation of radial artery with fingers. **(C)** Local anesthesia with lidocaine. **(D)** Puncture TRA with 20 G plastic cannula. **(E)** Mini guidewire advancement. **(F)** Radial artery sheath insertion. **(G)** An air power device is placed over the puncture site with the sheath retracted. **(H)** Sheath removal while rotating pneumatic screw until hemostasis was achieved. **(I)** Evaluation of radial patency by Doppler ultrasound.

In the TRA group, the puncture site was located at proximal 2 cm of styloid and the procedure was consistent with DRA (Figures 2A–G). Patent hemostasis was achieved by a radial compression device for 4–6 h (Air Power, Shenzhen, China; Figure 2H). Before discharge, radial artery patency was evaluated with ultrasound (Figure 2I).

### Study endpoint and definition

The primary endpoint was the rate of forearm RAO, assessed by an independent clinical operator using Doppler ultrasound before discharge. Secondary endpoints included access success rate, puncture time, fluoroscopy time, dose area product, air kerma, contrast volume, procedure time, haemostasias time and access site complications such as hematoma, perforation, local numbness, pseudoaneurysm, and arteriovenous fistula.

Radial artery occlusion was defined as the absence of anterograde flow on color Doppler ultrasound and the absence of a pulse wave on pulsed Doppler ultrasound. Access success occurred when an introducer sheath can be properly placed through the initial puncture artery (11). The puncture time was defined as the time interval between local anesthesia induction and successful sheath insertion (11). Door-to-wiring time was expressed as the duration from hospital arrival to guidewire passage through the culprit lesion (19). Haemostasias time was defined as the period from the application of the compression band to its removal.

### Sample size and statistical analysis

Based on results of previous studies, the sample size was calculated to establish superiority of DRA in preventing forearm RAO. The incidence of forearm RAO was approximately 10% (20–22) and 1% (14, 23) for the TRA and DRA group, respectively. Using the PASS 15.0 software, we calculated the sample size of 97 patients for per group, with statistical power of 80% and an alpha error of 5%.

Normally distributed continuous variables were reported as mean ± standard deviation and compared using Student’s *t*-test, while non-normally distributed continuous variables were presented as median (interquartile range) and compared using the Mann–Whitney *U* test. Categorical variables were expressed as frequencies and percentages. Pearson’s chi-squared test or Fisher’s exact test was used to analyze categorical variables, as appropriate. The primary endpoint (the rate of forearm RAO before discharge) were evaluated using an intention-to-treat (ITT, on the basis of randomization allocation to either the TRA or the DRA group) and a per-protocol analysis (PP, considering patients who ended up in either group). For the secondary endpoint, the analyses were performed on the ITT population. Potential risk factors for forearm RAO were investigated by univariate and multivariable logistic regression models. All variables with *p* < 0.1 in univariable analysis were entered in the multivariable model. Statistical analyses were performed using SPSS software v.24.0 (IBM, Armonk, NY, USA). Statistical significance was set at *P* < 0.05.

## Results

**FIGURE 3:**
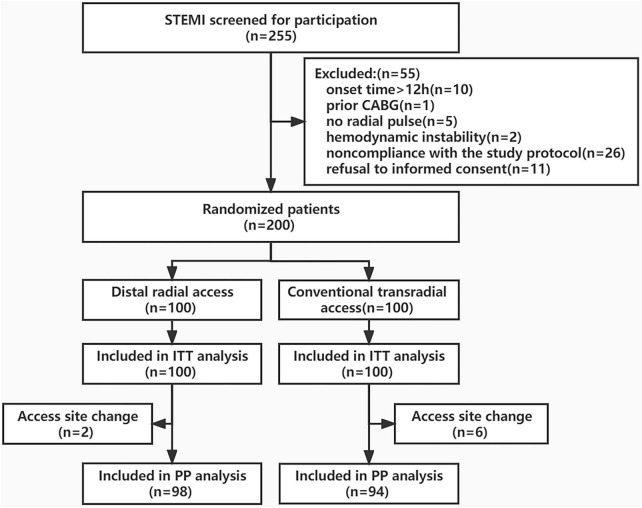
Study flowchart. STEMI, ST-segment elevation myocardial infarction; CAGB, coronary artery bypass graft; ITT, Intention-to-Treat; PP, Per-protocol.

From January 2022 to September 2022, 255 eligible patients with STEMI were recruited for participation, of which 55 were excluded. The primary reasons for exclusion were as follows: prior CABG (*n* = 1), no radial pulse (*n* = 5), hemodynamic instability (*n* = 2), onset time > 12 h (*n* = 10), non-compliance with the study protocol (*n* = 26), and refusal to provide informed consent (*n* = 11). Two hundred STEMI patients were randomized to either the DRA group (*n* = 100) or the TRA group (*n* = 100). The flowchart of the study is shown in Figure 3.

### Patient baseline and procedural characteristics

The average age of the patients assigned to the DRA and TRA groups was 61.6 ± 12.0 and 60.9 ± 13.5 years, respectively, with a male predominance in both groups (87.0 vs. 80.0%, *P* = 0.182). Previous intervention through the right TRA was reported in 18.0% (*n* = 18) of the DRA group and 12.0% (*n* = 12) of the TRA group; DRA was formerly used in only 1.0% (*n* = 1) of the DRA group. The baseline characteristics did not differ significantly between the groups (Table 1). Multivessel disease was observed more frequently in the TRA group than in the DRA group (*P* < 0.001). Table 2 summarizes the procedural characteristics.

**TABLE 1:** Patients’ baseline characteristics.

Variables	DRA (*n* = 100)	TRA (*n* = 100)	*P*-value
Age (years)	61.6 ± 12.0	60.9 ± 13.5	0.688
Male, no. (%)	87 (87.0)	80 (80.0)	0.182
BMI (kg/m^2^)	25.4 (24.2–27.1)	25.9 (23.6–28.7)	0.241
**Cardiovascular risk, no. (%)**			
Hypertension	55 (55.0)	61 (61.0)	0.390
Diabetes mellitus	26 (26.0)	18 (18.0)	0.172
Dyslipidemia	31 (31.0)	33 (33.0)	0.762
Current smoking	56 (56.0)	52 (52.0)	0.570
Chronic renal failure	3 (3.0)	1 (1.0)	0.621
Atrial fibrillation	1 (1.0)	2 (2.0)	1.000
New-onset of AF	2 (2.0)	5 (5.0)	0.445
Peripheral arterial disease	5 (5.0)	5 (5.0)	1.000
Previous stroke	12 (12.0)	9 (9.0)	0.489
Prior DRA	1 (1.0)	0	1.000
Prior TRA	18 (18.0)	12 (12.0)	0.235
LVEF (%)	62.0 ± 10.3	60.0 ± 9.9	0.162
Killip ≥ 2, no. (%)	12 (12.0)	13 (13.0)	0.831
**Vital signs**			
SBP (mmHg)	120.2 ± 19.7	115.2 ± 18.9	0.071
DBP (mmHg)	78.7 ± 15.6	76.7 ± 16.1	0.385
HR (beats/min)	73.7 ± 16.1	79.8 ± 19.9	0.018
**In-hospital medications, no. (%)**			
Aspirin	98 (98.0)	99 (99.0)	1.000
Clopidogrel	19 (19.0)	18 (18.0)	0.856
Ticagrelor	80 (80.0)	82 (82.0)	0.718
ACEI/ARB	76 (76.0)	77 (77.0)	0.868
Beta-blocker	70 (70.0)	82 (82.0)	0.047
Statin	96 (96.0)	95 (95.0)	1.000

BMI, body mass index; AF, atrial fibrillation; DRA, distal radial artery; TRA, conventional transradial access; LVEF, left ventricular ejection fraction; SBP, systolic blood pressure; DBP, diastolic blood pressure; HR, heart rate; ACEI, angiotensin converting enzyme inhibitor; ARB, angiotensin receptor blocker.

**TABLE 2:** Procedural characteristics and outcomes.

Variables	DRA (*n* = 100)	TRA (*n* = 100)	*P*-value
Primary PCI loading, no. (%)			0.637
Aspirin 300 mg + Clopidogrel 600 mg	9 (9.0)	11 (11.0)	
Aspirin 300 mg + Ticagrelor 180 mg	91 (91.0)	89 (89.0)	
**Medications administered during procedure, no. (%)**			
GP IIb/IIIa inhibitor	7 (7.0)	13 (13.0)	0.157
Antithrombin			0.339
Bivalirudin	76 (76.0)	70 (70.0)	
Heparin	24 (24.0)	30 (30.0)	
**Location of myocardial infarction, no. (%)**			
Anterior	45 (45.0)	43 (43.0)	0.776
Inferior	53 (53.0)	55 (55.0)	0.777
Lateral or other	2 (2.0)	2 (2.0)	1.000
**Infarct-related vessel, no. (%)**			
LAD	49 (49.0)	45 (45.0)	0.671
LCX	8 (8.0)	10 (10.0)	0.637
RCA	42 (42.0)	45 (45.0)	0.776
LM	1 (1.0)	0	1.000
**Cardiac catheterization procedure, no. (%)**			
PCI performed	96 (96.0)	100 (100.0)	0.121
Stenting performed	73 (73.0)	80 (80.0)	0.243
Drug-eluting balloons	9 (9.0)	9 (8.0)	1.000
No. of catheters per patient	1.2 ± 0.4	1.2 ± 0.5	0.667
BAT	1 (1.0)	1 (1.0)	1.000
IABP	0	2 (2.0)	0.497
OCT	53 (53.0)	57 (57.0)	0.570
ACT (s)	363 (293–432)	363 (304–417)	0.994
**Angiographic results, no. (%)**			
Multivessel disease	62 (62.0)	84 (84.0)	< 0.001
Multivessel PCI	5 (5.0)	11 (11.0)	0.118
**TIMI flow at baseline**			
0/1	82 (82.0)	89 (89.0)	0.160
2	12 (12.0)	9 (9.0)	0.489
3	6 (6.0)	2 (2.0)	0.279
**TIMI flow after procedure**			
0/1	3 (3.0)	1 (1.0)	0.621
2	0	3 (3.0)	0.246
3	97 (97.0)	96 (96.0)	1.000

PCI, percutaneous coronary intervention; LAD, left anterior descending artery; LCX, left circumflex artery; RCA, right coronary artery; LM, left main; BAT, balloon-assisted tracking; Glycoprotein, IIb/IIIa; IABP, intra-aortic balloon pump; OCT, optical coherence tomography; ACT, activated clotting time; TIMI, Thrombolysis in Myocardial Infarction.

### Arterial access-related outcomes

**FIGURE 4:**
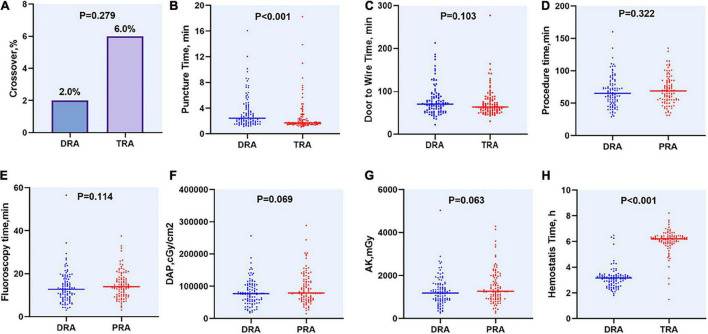
Difference of access site outcomes between two groups. **(A)** Crossover of access site, **(B)** Puncture time, **(C)** Door to Wire time, **(D)** Procedure time, **(E)** Fluoroscopy time, **(F)** DAP, **(G)** AK, **(H)** Hemostasis time. DAP, dose area product; AK, air kerma.

The rates of access success in the DRA and TRA groups were comparable (98.0 vs. 94.0%, *P* = 0.279). Two patients were converted to TRA in the DRA group, whereas four patients were successfully converted to DRA, one patient to femoral access, and another to brachial artery access in the TRA group. The main reasons for the access site crossover are listed in Table 3. Left side access was chosen in one person of DRA group due to right RAO. The puncture time was longer in DRA than in TRA [2.4 (1.7–4.2) min vs. 1.7 (1.4–2.3) min; *P* < 0.001], whereas the door-to-wire time was not significantly different between the two groups [71 (54–88) min vs. 64 (56–82) min; *P* = 0.103]. Hemostasis time was noticeably shorter in the DRA group than in the TRA group [3.1 (2.7–3.3) h vs. 6.2 (5.9–6.4) h; *P* < 0.001]. Total fluoroscopy dose area product and air kerma of DRA group were decreased compared TRA group but the difference was not significant (*P* = 0.066, *P* = 0.061), respectively. However, no significant differences were reported in the procedure time, fluoroscopy time and contrast volume (Table 3 and Figure 4).

**TABLE 3:** Primary and secondary outcomes.

Variables	DRA (*n* = 100)	TRA (*n* = 100)	*P*-value
Right access side	99 (99.0)	100 (100.0)	1.000
Access success, no. (%)	98 (98.0)	94 (94.0)	0.279
Crossover of access site, no. (%)	2 (2.0)	6 (6.0)	
Failure artery puncture	1	4	
Failure insertion of guide wire	1	2	
Puncture time (min)	2.4 (1.7–4.2)	1.70 (1.4–2.3)	<0.001
Door-to-wire time (min)	71 (54–88)	64 (56–82)	0.103
Procedure time (min)	65 (50–79)	69 (55–83)	0.322
Fluoroscopy time (min)	12.7 (8.7–17.5)	13.9 (9.9–17.8)	0.114
DAP (cGy/cm^2^)	76811 (53450–103068)	78751 (59466–119160)	0.069
AK (mGy)	1179.6 (855.8–1569.4)	1263.3 (930.2–1962.3)	0.063
Contrast volume (ml)	170 (112–210)	170 (130–210)	0.248
Selective compression closure device, no. (%)	93 (93.0)	95 (95.0)	0.552
Hemostasis time (h)	3.1 (2.7–3.3)	6.2 (5.9–6.4)	<0.001
Hospital stay (day)	6 (5–7)	6 (5–7)	0.654
**Complications, no. (%)**			
**Hematoma**			
≤ 5 cm	4 (4.0)	6 (6.0)	0.516
> 5 cm	0	6 (6.0)	0.029
Local numbness	1 (1.0)	0	1.000
Forearm RAO	2 (2.0)	9 (9.0)	0.030
Pseudoaneurysm	0	1 (1.0)	1.000
Arteriovenous fistula	0	0	−

DAP, dose area product; AK, air kerma; RAO, radial artery occlusion.

### Primary endpoint

**FIGURE 5:**
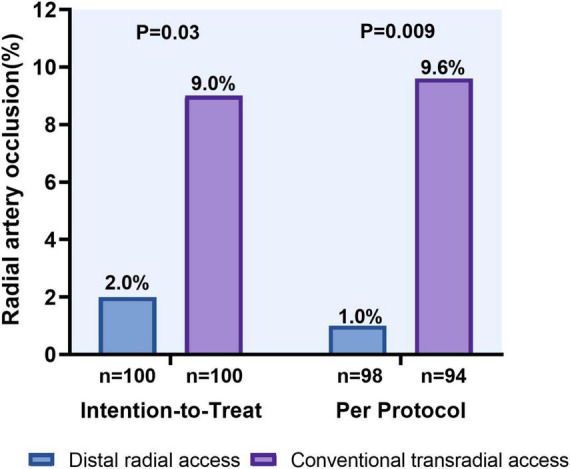
Forearm radial artery occlusion rates using the distal versus conventional transradial access.

Doppler evaluation of the radial artery was performed in all patients. The length of hospital stay was 6 (5–7) days in both groups. In an ITT analysis, DRA was superior to TRA in terms of the forearm RAO rate [2 (2.0%) vs. 9 (9.0%), *P* = 0.030]. Likewise, the reduction in forearm RAO in the DRA group was even more prominent in a per-protocol analysis [1 (1.0%) vs. 9 (9.6%); *P* = 0.009] (Figure 5). Logistic multivariate analysis showed that hemostasis time was identified as independent risk factors for forearm RAO with an area under curve (AUC) of 0.787 (95% CI 0.689–0.885, *P* = 0.001) (Table 4).

**TABLE 4:** Logistic regression analysis of forearm radial artery occlusion.

Variable	Univariate analysis			Multivariable analysis		
	*P*-value	OR	95% CI	*P*-value	OR	95% CI
Male	0.081	0.317	0.087–1.153	–	–	–
Peripheral arterial disease	0.061	0.199	0.037–1.076	–	–	–
Hemostasis time	0.007	0.436	0.237–0.800	0.017	0.379	0.171–0.842
Transradial access	0.047	0.206	0.043–0.981	–	–	–

### Complications

**FIGURE 6:**
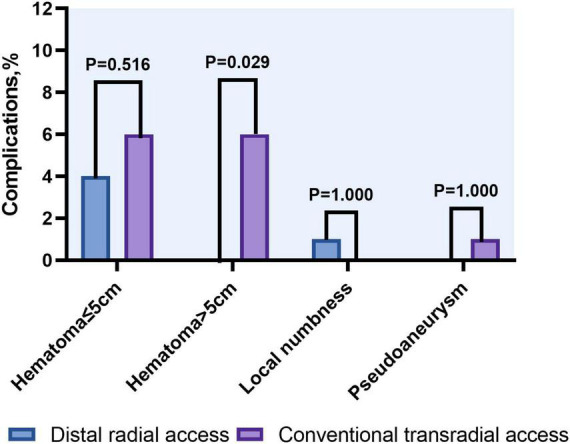
Access site complications between distal and conventional transradial access.

Local hematoma ≤ 5 cm in diameter was observed in four patients in the DRA group and six patients in the TRA group, while those > 5 cm were significantly more frequent in the TRA group (0 vs. 6.0%, *P* = 0.029). Pseudoaneurysm occurred in one patient (1.0%) in the TRA group and recovered after 1 week of conservative treatment. Local numbness was identified in one patient in the DRA group within 24 h post-procedure (Table 3 and Figure 6).

## Discussion

This is the first randomized study to evaluate the feasibility and safety of DRA and TRA for primary PCI in patients presenting with STEMI. We demonstrated that DRA was associated with analogous rate of successful puncture, decreased rate of forearm RAO, shorter hemostasis time, and lower rate of vascular complications when compare with TRA. Although the puncture time was higher in the DRA, the door-to-wire and procedure time were similar to those in the TRA.

Previous studies have shown the high success rate of DRA for CAG or PCI, ranging from 70 to 100% (24–27). Interestingly, only few STEMI patients were enrolled in these studies because of the time-sensitive procedure for primary PCI. Recently, Kim et al. (28) recruited 138 patients with STEMI in a retrospective observational study, and 92.8% of them punctured successfully *via* the DRA route. In another retrospective cohort study (29), all 30 patients were successfully punctured *via* the left DRA. Lee et al. (19) compared DRA, TRA, and femoral access in patients with STEMI undergoing primary PCI, and the success rate of DRA was 83.3% (35/42). In this study, the rate of access success in the DRA group was 98.0%. The following possible reasons that may help to explain such phenomenon: Firstly, DRA was used as default access in our center from December 2020, more than 1,000 CAG and intervention procedures were performed by 3 operators *via* DRA before our study. Secondly, previous study (30) showed using a spring-coiled wire has a higher incidence of failure. In our study, Radifocus Introducer II Standard kits (Terumo) including 0.021″ straight hydrophilic guide wire was used. In addition, PTCA guidewire was also used in some cases when mini guidewire failed to pass DRA. Thirdly, the limited sample size was another reason which led to high success rate bias of DRA.

Several studies comparing puncture time, defined as the interval from local anesthesia to sheath cannulation, have shown conflicting results between the DRA and TRA group. In the latest randomized controlled trial (DISCO RADIAL), Aminian A et al. (17) reported the longer puncture time was required in the DRA group than the TRA group [2 (1–4) min vs. 1 (1–3) min, *p* < 0.001]. Other studies have yielded the consistent results (13, 25, 31, 32). In contrast to above results, there was no difference in puncture time between two groups (14, 23, 26, 33). Besides, in a retrospective observational studies of STEMI patients, the puncture time *via* DRA was 2.7 ± 1.6 min (28). Lee OH et al. (19) presented the puncture time for primary PCI *via* DRA and TRA was 116.1 ± 56.1 s vs. 100.8 ± 46.0 s (*P* = 0.27), respectively. In our study, the puncture time was higher in the DRA group than in the TRA group [2.4 (1.7–4.2) min vs. 1.7 (1.4–2.3) min; *P* < 0.001]. Because of its special anatomy and prolonged puncture time, it is reasonable to suppose that this new access might delay door-to-wire time in patients with STEMI. However, no delay was observed in the present study. Nevertheless, DRA for primary PCI might be a feasible alternative for patients with STEMI.

The hemostasis time of TRA in our study (6.2 h) was similar to another study, stating that the longer hemostasis time in Asian was required than that in Europe and the USA and more Asian had a hemostasis time of more than 6 h (34). Conspicuously, hemostasis time in the DRA group was even shorter, about twice less than TRA group in our study. As previous reported, DRA was associated with a shorter hemostasis time (24, 25, 35, 36). Potential explained could be the distal radial artery’s slightly smaller diameter, as well as the support of the scaphoid and trapezium carpal bones. DRA presented lower total fluoroscopy dose area product and air kerma compared with TRA group, but the difference was not significant. Moreover, there was no difference between two groups in procedure time, fluoroscopy time, and contrast volume.

Radial artery occlusion remains the most frequent complication of transradial access, hindering repeated use of the radial artery for coronary artery bypass grafting, arteriovenous fistula creation for hemodialysis, and further catheterization (4). The incidence of RAO usually varies between 1 and 10%, but can be up to 30% (9). The DRA has served as an alternative access for cardiac catheterization (35, 37), yielding the potential for highly reduced RAO. Due to the dual blood supply of the hand, even slowing or occlusion of the DRA does not affect the anterior flow of the forearm radial artery (10). A single-center, larger randomized trial involving 1,042 consecutive patients (13), showed that a difference in terms of the forearm RAO by Doppler ultrasound at 60 ± 30 days follow-up, which was significantly reduced in the DRA group (3.7 vs. 7.9%, *P* = 0.014). Furthermore, the reduction in forearm RAO at 24 h after the procedure in the DRA group was even more prominent than that in the TRA group in the DAPRAO trial by Eid-Lidt (0.7 vs. 8.4%, *P* = 0.002) (14). Nevertheless, the recently released DISCO RADIAL trial (17) showed no significant difference in RAO rates between the two groups (0.31 vs. 0.91%, *P* = 0.29). The extreme incidence of RAO would be explained according to adequate anticoagulation, effective spasmolytic treatment, as well as assuring extensive operational experience with both access methods in this study. All of STEMI patients in aforementioned researches were excluded. Our randomized study showed that DRA significantly reduced forearm RAO rate compared to TRA (2.0 vs. 9.0%, *P* = 0.030) in primary PCI.

Regarding other complications, although no difference was observed between two groups in local hematomas (≤ 5 cm), the rate of hematomas (> 5 cm) was lower in DRA than in TRA patients, similarly agreed in another study (13). It should be noted that local numbness occurred in one patient of DRA group since the radial nerve is close to the radial artery in the anatomical snuffbox. This rate was reported approximately 1% in previous study (28, 38, 39).

This study had several limitations. It was a single-center study, with a relatively small number of patients. Moreover, all procedures were performed by three experienced operators, restricting extrapolation of our results to less experienced centers. Finally, nearly 90% of patients presented with Killip 1 class and only 2 out of 255 patients screened were excluded because of hemodynamic instability. Moreover, most patients were males and with a normal BMI. Accordingly, our results only apply to a selected portion of STEMI patients with stable clinical conditions. A large-scale, multicenter, randomized study is needed to compare the feasibility and safety of DRA with TRA group in STEMI patients undergoing primary PCI.

## Conclusion

The use of DRA prevented forearm RAO before discharge, shortened hemostasis time, and reduced complications compared to TRA. With certain manipulative skills and rational choice of wires, DRA is a feasible and safe alternative for primary PCI in patients with STEMI.

## Data availability statement

The raw data supporting the conclusions of this article will be made available by the authors, without undue reservation.

## Ethics statement

The studies involving human participants were reviewed and approved by the Ethics Committee of Beijing Luhe Hospital, Capital Medical University. The patients/participants provided their written informed consent to participate in this study.

## Author contributions

YJW, ZJL, and JCG contributed to the conception and design of the study. YJW, YXW, ZXL, YTW, SHW, RX, LBZ, and YPW conducted the single center clinical trial and collected the data. YJW, ZJL, and YXW analyzed data and wrote the first draft of the manuscript. JCG contributed to the manuscript revision. All authors contributed to the article and approved the submitted version.

## Abbreviations

TRA: conventional transradial access
DRA: distal radial access
RAO: radial artery occlusion
STEMI: ST-segment elevation myocardial infarction
PCI: percutaneous coronary intervention.
